# Performance analysis of EAC joint GMP inspections (2016–2022): a pathway to strengthening regulatory systems and building capacity in Africa’s less resourced authorities

**DOI:** 10.3389/fmed.2025.1644446

**Published:** 2025-09-17

**Authors:** Jane Mashingia, Noel Aineplan, Kari Clase, Steven Bryn, Zita Ekeocha

**Affiliations:** ^1^African Union Development Agency, New Partnership for Africa’s Development (AUDA-NEPAD), Johannesburg, South Africa; ^2^Manager International Affairs, National Drug Authority (NDA), Kampala, Uganda; ^3^Department of Agricultural and Biological Engineering, Purdue University, West Lafayette, IN, United States; ^4^Biotechnology Innovation and Regulatory Science (BIRS) Center, West Lafayette, IN, United States

**Keywords:** Good Manufacturing Practices (GMP), Joint inspections, East African Community (EAC), National Regulatory Authorities (NRAs), African Medicines Agency (AMA), Corrective and Preventive Actions (CAPA), Regulatory Harmonisation and Capacity Building

## Abstract

**Introduction:**

National Regulatory Authorities have the responsibility of ensuring that pharmaceutical manufacturers comply with Good Manufacturing Practices (GMP) to ensure that medicinal products are consistently produced according to quality standards. Since July 2016, the EAC National Medicines Regulatory Authorities (NRAs) have been collaborating to conduct joint Good Manufacturing Practice (GMP) inspections. This study was conducted to (a) assess the joint GMP inspection procedure, (b) determine key milestones and target timelines, (c) identify areas of improvement and opportunities for capacity building, and (d) identify the contribution of the EAC joint GMP inspections in the operationalization of the African Medicines Agency (AMA).

**Methods:**

A retrospective review of the timelines for joint GMP inspections conducted from 2016 to 2022 was performed using data recorded in the EAC metric tool. Data is captured based on key milestones, including screening, scheduling joint GMP inspections, planning for joint GMP inspections, conducting physical inspections, reviewing GMP documentation, writing inspection reports, peer reviewing inspection reports, reviewing CAPAs, communicating with applicants, and issuing GMP compliance certificates. JMP was used as the statistical software to analyze the main milestones of the EAC joint GMP inspection process. The study also looked into the twinning model and joint inspections for capacity building among NRAs.

**Results:**

Results indicated that a total of 37 pharmaceutical manufacturing facilities, located in Africa, Asia, and Europe, were jointly inspected (35 physical inspections and two desk reviews) between 2016 and 2022. The inspected facilities manufacture vaccines, non-beta-lactam tablets/capsules, sterile products, and other biologicals. Of the 37 facilities inspected, 65% (24) met EAC GMP standards and received a certificate of compliance, valid for 3 years. 8% (3) of the facilities inspected between 2018 and 2022 failed to meet GMP standards and were advised to apply for reinspection. Two facilities have not submitted corrective and preventive action (CAPA) since 2016, and three facilities have not submitted one since 2022. The study further revealed that one facility is pending joint inspection, one has been awaiting CAPA review since 2019, and one with a CAPA is still under review. Two facilities scheduled for physical inspection in 2020 could not be inspected due to COVID-19 travel restrictions. Timelines for key milestones of the joint GMP inspection process showed that overall scheduling of joint GMP inspections took more than 14 workdays, such as 57 days in 2018, 24 days in 2019, 43 days in 2020, and 28 days in 2022. Median times for planning EAC joint GMP inspections aligned with the set timelines of 30 days; however, in 2017, the median duration was 40 days. All physical inspections conducted between 2016 and 2022 were completed within the median timeline of 3 days. Nonetheless, the median time for GMP report writing in 2016 was 16 days, slightly exceeding the established timeline of 14 days. Findings also indicated that manufacturing facilities (Applicants) take a lengthy time to submit CAPA, which should be done within 90 days. Median submission times for CAPA were 135 days in 2018 and 125 days in 2019. In 2022, notable improvements were observed, with applicants submitting CAPA in a median time of 49 days.

**Discussion:**

EAC GMP inspections have served as a model for building capacity in lessresourced NRAs through the twinning program. The joint inspections are carried out by two inspectors from more experienced National Regulatory Authorities (NRAs) and one inspector from less-resourced NRAs. These NRAs benefit from learning and gaining practical skills, while also building confidence and trust among themselves. This model is suitable for adoption at the continental level by the African Medicines Agency and the African Medicines Regulatory Harmonization Programme to support reliance, convergence, and harmonization. The study identified several gaps, including incomplete data in the metric tool and a lack of monitoring and recording timelines for joint GMP desk assessments. Based on the findings, the EAC region needs to establish an annual planning mechanism for joint GMP inspections and update the metric tool to include key milestones and timelines for the joint GMP desk assessment process. Enhancing the stakeholders’ feedback mechanism is also essential for reducing delays in submitting corrective and preventive actions (CAPA) from facilities and shortening timelines for the joint regulatory inspection process.

## 1 Introduction

The EAC network of the National Medicines Regulatory Authorities (NRAs) is responsible for ensuring that marketing authorization holders manufacture medicinal products in line with Good Manufacturing Practices (GMP) requirements to avoid exposing patients to risks due to insufficient safety, quality, or efficacy (1, 2). The GMP covers all aspects of production personnel, premises, facilities, materials, documentation, and quality control. The entire process of manufacturing medicinal products—from the supply of raw materials to the release of finished products for distribution—is subject to GMP requirements (3–5). Since July 2016, the EAC has conducted 37 inspections of pharmaceutical and vaccine-manufacturing facilities within the framework of the EAC-MRH program. The EAC-MRH program was the first pilot project of the African Medicines Regulatory Harmonization initiative, which involved seven National Medicines Regulatory Authorities (NRAs) collaborating in various regulatory functions and set the foundation for establishment African Medicines Agency (AMA) (6–9).

### 1.1 Overview of the EAC national medicines regulatory authorities

Table 1 provided an overview of the Seven National Medicines Regulatory Authorities (NRAs) which participated in the EAC Joint GMP inspections namely, National Drug Authority (NDA)-Uganda, Pharmacy and Poisons Board (PPB)-Kenya, Drug and Food Control Authority (DFCA)-South Sudan, Aurorite Burundaise de Regulation des Medicaments (ABREMA)-Burundi, Rwanda Food and Drugs Authority (Rwanda FDA) and Tanzania Medicines and Medical Devices Authority (TMDA) and Zanzibar Food and Drug Agency (ZFDA)-United Republic of Tanzania. TMDA and Rwanda FDA reached WHO maturity level 3 (ML3) for the regulation of medicines and vaccines (non-producing) in April 2019 and December 2024, respectively. Other participating NRAs are in the process of implementing their institutional development plans (IDPs) at different levels.

**Table 1:** Overview of the EAC national medicines regulatory authorities.

Characteristic	Burundi (ABREMA)	Kenya (PPB)	South Sudan (DFCA)	Uganda (NDA)	Rwanda (Rwanda FDA)	Tanzania (TMDA)	Zanzibar (ZFDA)
Population (in millions)	13	55.65	13	50	13.2	61.7	1.8
Agency Staff	33	195	40	400		350	176
Number of GMP Inspectors	2	31	3	37	12	92	10
Total GMP Applications received between 2023 to 2024	0		15	337		230	7

The National Drug Authority (NDA) of Uganda is the main agency responsible for receiving and processing applications for EAC joint GMP inspections. The NRAs of the Democratic Republic of Congo (DRC) and Somalia were not included in the study because they joined the East African Community in July 2022 and March 2024, respectively. Both countries are working on integrating into EAC projects and programs. Among all EAC NRAs, TMDA, NDA, and PPB receive more applications compared to DFCA, the Rwanda FDA, and the ZFDA. Joint inspections have taken place for common applications drawn from seven participating NRAs. However, the metric tool did not define applications for joint inspections initiated by joint assessment procedures or those identified through the mapping of common applications in seven NRAs.

Joint inspections to assess compliance with GMP have contributed to cost savings for pharmaceutical manufacturers and NRAs, reduced the duplication of efforts, and promoted reliance, convergence, and collaboration between the EAC NRAs (10–12). Less-resourced NRAs have also benefited from joint inspections as part of capacity building to strengthen their competency, skills, and knowledge regarding the inspection of facilities for manufacture of finished pharmaceutical products and biotherapeutic manufacturing industries (13, 14).

**Figure 1:**
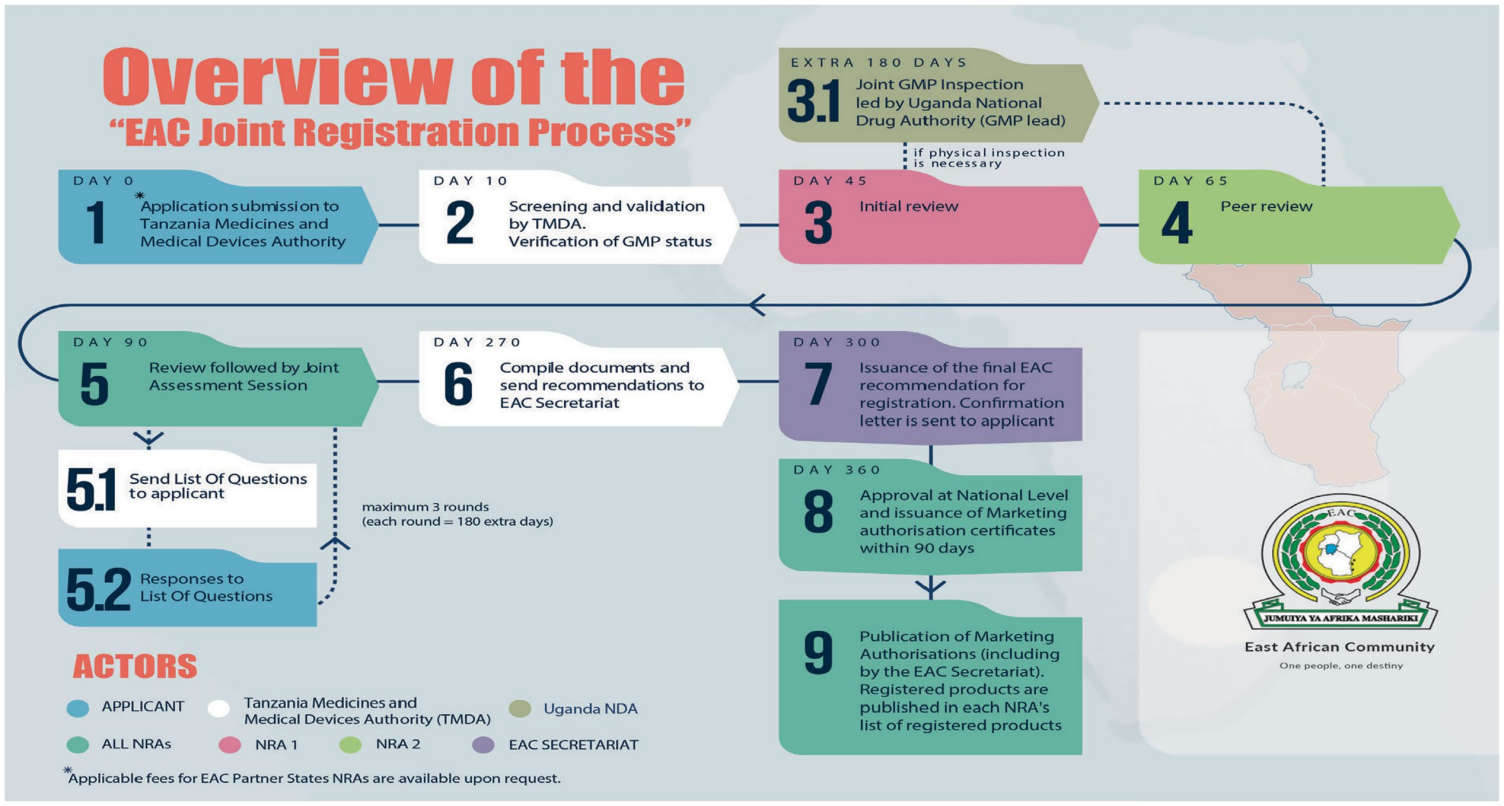
EAC joint assessment procedure of medicinal product dossiers.

**Figure 2:**
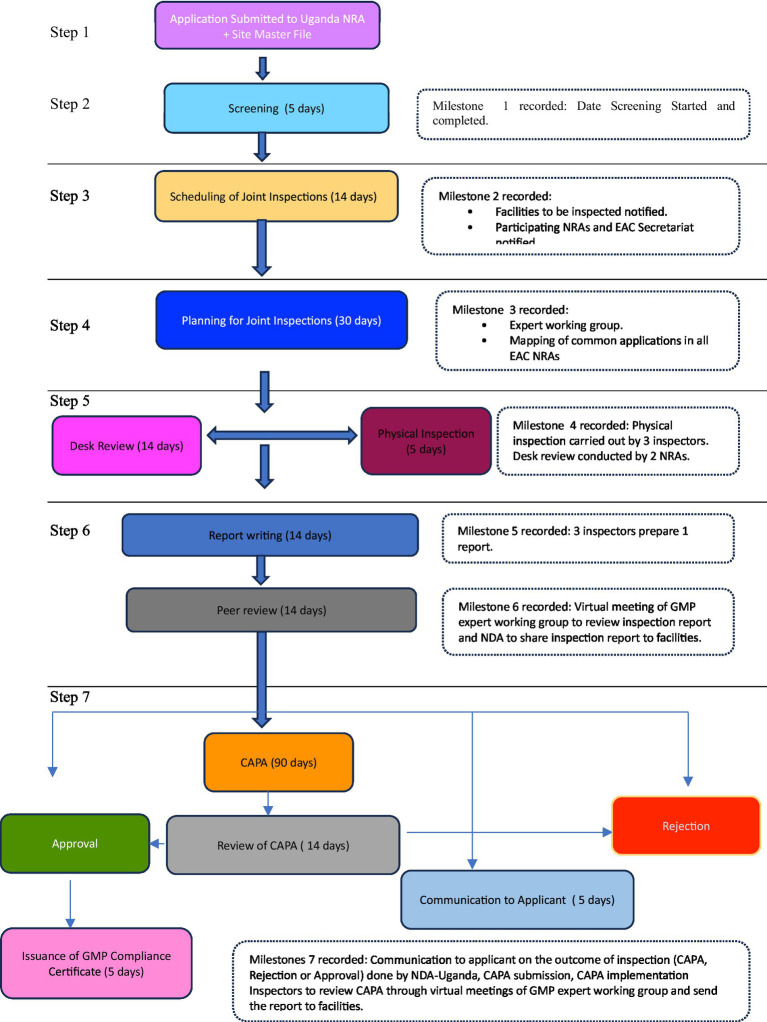
Process map of EAC joint GMP inspections.

Domestic and foreign pharmaceutical manufacturers of medicinal products to be marketed in the EAC region are subjected to GMP conformity assessments and must meet acceptable standards, as stipulated in the harmonized guidelines (1). Joint inspections, which are part of the joint assessment procedure (Figure 1), involve the physical inspection of facilities or a desk assessment of GMP documentation. It is a stepwise approach, and each step has a timeline provided in the metric tool and client service charter of the EAC-MRH program (15). A metric tool was used to record the administrative information of the facility and timelines for each milestone, as shown in Figure 2.

Analysis of timelines for EAC joint inspections has not been conducted since the commencement of the regional regulatory pathway; however, timelines for various milestones of the EAC joint scientific assessment of medicinal product dossiers were analyzed from 2018 to 2021. The findings indicated that the overall median approval times (joint scientific assessment to regional recommendation) surpassed the 465-day target, while the median time to issue marketing authorization by EAC NRAs following EAC joint assessment was more than the 116-day target (16). Some EAC joint GMP inspections conducted between 2016 and 2022 were triggered through applications for the joint assessment of medicinal product dossiers. Nonetheless, limited information is available on the performance, predictability, and efficiency of the joint inspection procedure and the capacity building of less-resourced NRAs.

Therefore, this study constitutes the second part of an analysis of the timelines for the EAC joint regulatory review pathway to assess its performance, efficiency, and capacity. It examined the timelines for each milestone of the joint GMP inspections and identified areas for improvement and capacity development. The first analysis focused on the timelines for the joint assessment procedure and the findings indicated median overall approval times exceeding the EAC 465-day target and median times to issue marketing authorization following EAC joint assessment recommendation exceeded by 116-day target (16).

### 1.2 Overview of EAC joint GMP inspection procedure

Joint GMP inspections may occur through four mechanisms (17): (a) an application for the joint assessment of medicinal product dossiers, which requires a joint inspection of the manufacturing facility; (b) similar applications for marketing authorization of drug product to more than one NRA; (c) an official request from a manufacturer; and (d) a joint interest of at least two EAC Partner States NRAs (Table 2).

**Table 2:** Overview of fees applicable for GMP inspections.

Fees for GMP inspections (USD)							
Characteristic	Burundi (ABREMA)	Kenya (PPB)	South Sudan (DFCA)	Uganda (NDA)	Rwanda (Rwanda FDA)	Tanzania (TMDA)	Zanzibar (ZFDA)
East Africa	3,000	4,000	3,000	5,000	1,800	4,000	4,000
Rest of Africa	4,000	4,000	4,000	6,000	2,400	5,000	5,000
Rest of the World	6,000	4,000	6,000	8,000	NA	NA	NA
SADC	NA	NA	NA	NA	NA	4.500	4,500
Asia	NA	NA	NA	NA	3,000	6,000	7,000
Australia & New Zealand	NA	NA	NA	NA	3,600	7,000	8,000
America	7,000	NA	NA	NA	4,200	7,000	8,000
Europe	NA	NA	NA	NA	3,600	6,500	7,000

Figure 2 summarizes the steps involved in the joint GMP inspection procedure. The first step requires the applicant to submit an application, including the site master file and applicable fees, to the lead NRA for GMP, which is the National Drug Authority (NDA) of the Republic of Uganda. Additionally, the applicant must pay fees to other NRAs in accordance with their national fee structure (Step 1). Step 2 involves screening the application and site master using the NDA within five working days. The NDA scheduled joint inspections as highlighted in Step 3, with a maximum of three inspectors per site. These inspectors were drawn from mature regulatory authorities and a less resourced NRA. The participating NRAs and EAC Secretariat were notified of scheduled inspections by the NDA within 14 days. In Step 4, joint GMP inspections were planned through regional meetings of experts, in which common applications pending GMP inspections in all EAC NRAs were mapped. Sites for joint inspections are identified, and the NDA will seek consent from facilities to conduct joint inspections instead of individual country inspections. All the activities for Step 4 are completed within 30 days.

Step 5 involves physical inspection of the facility, which is conducted within 3–5 days, while desk assessment of GMP documentation is carried out within 14 days. Applications that qualify for desk assessment are mainly for facilities that have already been inspected and have valid GMP certificates offered by stringent regulatory authorities such as the United States Food and Drug Administration (US FDA), Health Canada, European Medicines Agency, Ministry of Health, Labor and Welfare of Japan, WHO prequalification program, WHO-Listed Authority, United Kingdom Medicines and Healthcare Products Regulatory Agency, and European Union Member States (18, 19). A desk assessment is conducted by one inspector from each of the two selected EAC NRAs.

Step 6 involves report writing. For physical inspection, three inspectors who conducted the joint inspection are required to prepare a report within 14 days after completion of the inspection. For a joint desk review, two inspectors prepare a report within 14 days. Subsequently, GMP expert meetings are convened by the EAC Secretariat for peer review of the inspection report, as indicated in Step 6. Virtual peer review meetings are conducted within 14 days, and inspectors will finalize draft reports incorporating input from other inspectors.

Step 7 is the last step, following the regional peer review meeting, which focuses on regional outcomes and recommendations. The expert working group recommends the approval or issuance of a certificate of GMP compliance if the facility meets the EAC GMP standards. The certificate is issued by the NDA on behalf of other EAC NRAs within 14 days. If the peer review meeting recommends that the facility prepare Corrective and Preventive Action (CAPA), the NDA shares the inspection report with the facility and requests that those concerned prepare and submit CAPA within 90 days. If the facility does not meet the EAC GMP standards, within 5 days, the NDA shares the inspection report and the outcomes of the peer review meeting, indicating that the facility has not met the GMP standards, then EAC NRAs reject it is not fit to produce medicinal products for human use.

After the applicant has submitted CAPA, it is reviewed by an expert working group, followed by physical inspections to verify if the facility has implemented corrective and preventive actions. Based on the findings, the facility may be approved and granted GMP certificates for compliance with the EAC GMP standards, or it may be rejected for failing to meet these standards.

### 1.3 Study objectives

The objectives of this study were as follows: (a) evaluate the EAC Joint GMP inspection procedure; (b) determine the key milestones and target timelines achieved in the joint inspection process; (c) gauge the general performance of the joint GMP inspections (physical and desk assessment); (d) identify areas for improvements and opportunities for capacity building and regulatory system strengthening; (e) understand contributions of EAC joint GMP inspections in the operationalization of the AMA.

## 2 Methods

### 2.1 Data collection process

The study utilized raw data collected from the EAC metric tool for joint GMP inspections conducted between 2016 and 2022. Analysis focused on the data collected between 2016 to 2022. Data were curated based on various milestones, including screening, scheduling of joint GMP inspections, planning for joint GMP inspections, physical inspections, desk review of GMP documentation, inspection report writing, peer review of inspection reports, review of CAPA, communication with applicants, and issuance of GMP compliance certificates.

### 2.2 Data analysis

Data from joint GMP inspections were analyzed using JMP. The analysis was based on the type of inspection conducted: desk review or physical inspection. The timelines for each milestone of the EAC joint GMP inspections were analyzed and explained as follows:

- Startup time (date of submission of application and master file, screening of the application, scheduling of joint inspections, communication to NRAs, communication to applicant, planning for inspection)
- Scientific inspection time (date of the desk assessment or physical inspection, report writing, peer review of the report, and regional recommendations)
- Communication of regional recommendation to the applicant (date when the regional recommendation reached the date the NDA communicated to the applicant on the outcome).
- Issuance of Certificate for Compliance to the EAC GMP standards by NDA
- Follow-up on CAPA implementation and verification (date of CAPA submission by the facility, review of CAPA by the expert working group, follow-up physical inspections to date, and regional recommendations).

### 2.3 Ethics approval

This study was approved by faculty members of the Biotechnology, Innovation and Regulatory Sciences Center. As the study did not involve human subjects, approval from Purdue University’s Human Research Protection Program and the Institutional Review Board (IRB) was not required. Furthermore, an application for any IRB in East Africa was not required, and the EAC NRAs agreed to conduct the research and publish the findings.

## 3 Results

### 3.1 Status of EAC joint GMP inspections conducted in 2016–2022

The metric tool included data from 45 facilities, including six in East Africa and India that received a one-year extension of their GMP certificate of compliance between 2020 and 2021. Due to the COVID-19 pandemic, physical joint inspections were paused, and the region lacked the technology and infrastructure to support remote inspections by NRAs and manufacturing sites. There was repetitive data entry for two facilities, and analysis of the remaining 37 facilities informed this study’s findings. The findings indicated that physical inspections were conducted in facilities located in Egypt, Tanzania, Morocco, India, China, France, Palestine, Bangladesh, Kenya, Moldova, Uganda, Italy, Germany, and Malawi.

Two of the 37 facilities manufacture veterinary products, while the remaining facilities produce human medicinal products, including vaccines, non-beta-lactam tablets and capsules, sterile products (injectable powders, solutions for injections, and eye drops), oral suspensions and liquids, biosimilars, creams, ointments, penicillin’s, and other biologicals.

The EAC has not yet established harmonized regulatory requirements, guidelines, or procedures for inspecting veterinary products. Veterinary facilities were inspected by the NDA in accordance with the procedure for recognizing regulatory decisions on GMP issued by other EAC NRAs (20), and the results of these inspections were adopted by other EAC NRAs.

**Figure 3:**
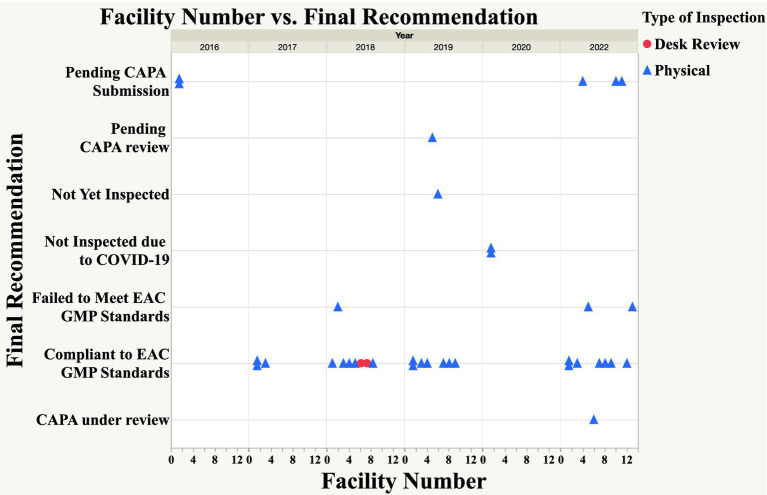
Status of EAC joint GMP inspections 2016–2022.

Based on the findings shown in Figure 3, 65% (24 of 37 facilities) of the facilities inspected through both physical and desk review met the EAC GMP standards and were issued a certificate of compliance, which is valid for 3 years. Three facilities inspected between 2018 and 2022 failed to meet the EAC GMP standards and were advised to apply for re-inspection. Two facilities have not submitted CAPA since 2016, and three have not submitted CAPA since 2022. The study also revealed that one facility is awaiting joint inspection, one has been waiting for review of CAPA since 2019, and another has their CAPA under review. Scheduled joint inspections of the two facilities did not take place in 2020 due to the COVID-19 pandemic.

A desk review of GMP documentation was conducted in 2018 for two applications, while in 2022, eight joint GMP desk assessments were carried out for facilities that met the criteria, in addition to 13 joint physical inspections, as shown in Figure 3 (21). Although the metric tool did not include data or timelines for the eight joint GMP desk assessments, the applicants were from Singapore, the United States, the Republic of Ireland, Portugal, Germany, Italy, Belgium, and Switzerland. According to the EAC procedure for joint GMP desk assessment (22), this process can be used to evaluate the GMP status of sites from countries with stringent drug regulatory authorities that may not undergo physical inspection due to existing mutual recognition agreements, after the initial mandatory GMP inspection or if the site is located in an exempted country.

### 3.2 Screening and scheduling timelines (startup time)

**Figure 4:**
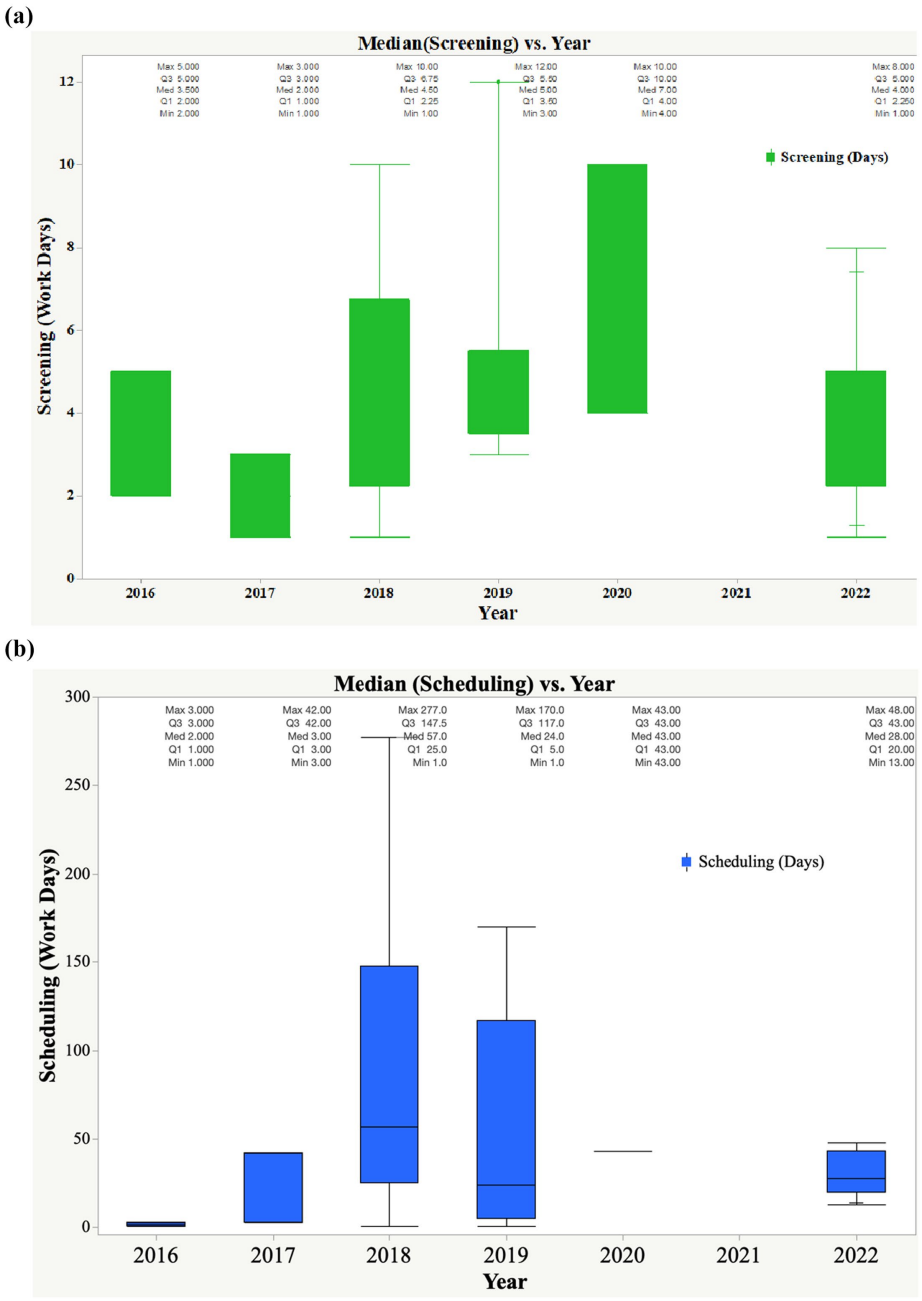
**(a)** Trend in median times for start-up (date of submission of application, master file, and screening of the application). **(b)** Trend in median time for scheduling of joint inspections.

Based on the EAC-MRH program client service charter and as shown in Figure 4, application documentation and master files were screened within five working days. The highest median timeline—7 days—was recorded in 2020, mainly due to the COVID-19 pandemic; the lowest median time—2 days—was observed in 2017. Applications and master files were screened, and sites were scheduled for joint GMP inspections; however, due to the lockdown, physical inspections could not take place. Based on the flow chart (Figure 2), EAC joint GMP inspections should be scheduled within 14 working days. However, the findings showed long median timelines for scheduling joint inspections—57 days in 2018, 24 days in 2019, 43 days in 2020, and 28 days in 2022.

### 3.3 Timelines for planning joint GMP inspections, communication with applicants/NRAs, and confirmation by applicants

**Figure 5:**
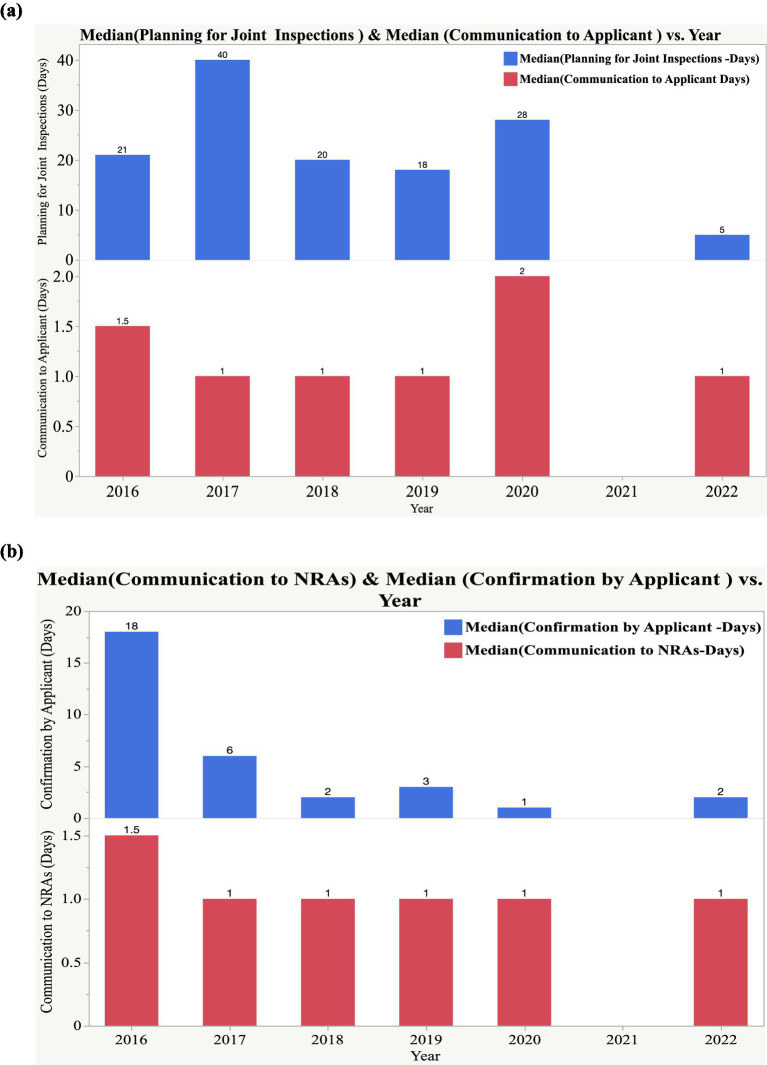
**(a)** Median timelines of planning for joint GMP inspections and communication to applicants. **(b)** Median timelines for communication to NRAs and confirmation by applicant.

The results indicated that the median time for planning EAC joint GMP inspections indicated consistency in adherence to the set timelines of 30 days. However, in 2017, the median time was 40 days, as indicated in Figure 5a. The planning phase involved experts from all the EAC NRAs through virtual or face-to-face workshops. The backlog of applications for GMP audits is normally mapped to identify common applications. A list of sites qualified for physical inspection and desk assessment is generated. Each matured NRA is assigned a site to inspect, and inspectors from less-resourced NRAs join the team based on twinning arrangements to build their knowledge and skills—ZFDA with PPB, DPML with TMDA, DFCA with NDA, and Rwanda FDA with NDA. Additionally, communication by the NDA to the applicant is maintained for 14 days. The NDA, as a lead agency in EAC joint GMP inspections, communicates with applicants to seek consent to inspect the facility through a joint procedure or inform the applicant of the planned joint inspection, especially when the joint assessment procedure requires a facility to be inspected. Furthermore, the NDA maintains constant communication with the other EAC NRAs, as shown in Figure 5b.

The applicant has a time frame of 30 days to confirm to the NDA the dates for joint inspections, and based on Figure 5b, all applicants for the period 2016–2022 had short turnaround times to confirm dates for inspections.

### 3.4 Timelines for joint regulatory inspections (desk assessment or physical inspections)

The timeline for conducting EAC joint physical inspections is three working days, whereas for the joint GMP, the desk assessment is 14 days. Three inspectors participating in physical inspections from matured and less-resourced NRAs are expected to prepare the report within 14 days of inspections. Peer review meetings (which are normally held virtually) involve other inspectors from the remaining EAC NRAs and are expected to provide feedback on the outcomes of the inspection within 14 days. The findings indicated that all physical inspections conducted between 2016 and 2022 were performed within a median timeline of 3 days. The median times for report writing were six (2016), one (2017), 8.5 (2018), four (2019), and 16 (2022) days, slightly higher than the set timeline of 14 days.

For the peer review of the report, the median timelines for 2016, 2019, and 2022 were 16, 33, and 15 days, respectively. The metric tool did not have data on peer reviews in 2016 and 2017. The inspection report was likely shared with the facilities once concluded by the three inspectors without peer review, as the joint inspection regulatory pathway was in the initial years of the pilot phase of the EAC-MRH program. The highest median timelines for peer review were observed in 2019 (33 days); however, a reduction in timelines was observed in 2022, with a median timeline of 15 days.

Based on the inspection outcomes, some facilities were found to be compliant with EAC GMP standards after physical inspections and were notified of the outcome; a certificate of compliance to GMP standards was issued by the NDA. In 2017, all three inspected facilities were compliant with GMP standards. Nevertheless, two facilities inspected in 2016 were requested to submit the CAPA; thus far, no submission has been made by the applicants.

**Figure 6:**
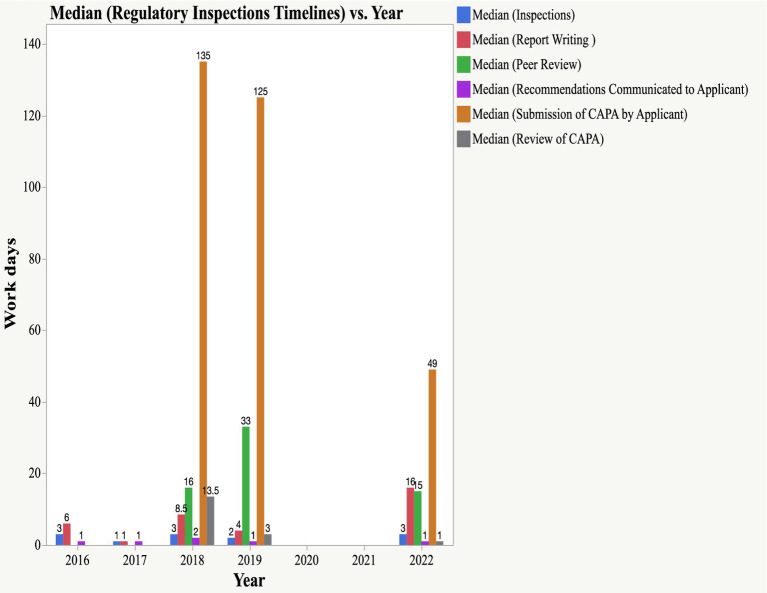
Timelines for Scientific Inspections (physical inspections, report writing, peer review, submission of CAPA and review of CAPA).

The findings indicate that applicants take a long time to submit a CAPA, which should be completed within 90 days. As shown in Figure 6, the median timelines for the submission of CAPA were 135 days (2018) and 125 days (2019). In 2022, more improvements were observed in CAPA submissions by applicants, with a median timeline of 49 days. EAC auditors reviewed CAPA within a set timeline of 14 days. The median timelines for the review of CAPA were 13 days, 3 days and 1 day in 2018, 2019, and 2022, respectively.

### 3.5 Median timelines for implementation of CAPA and communication on outcomes of CAPA review

**Figure 7:**
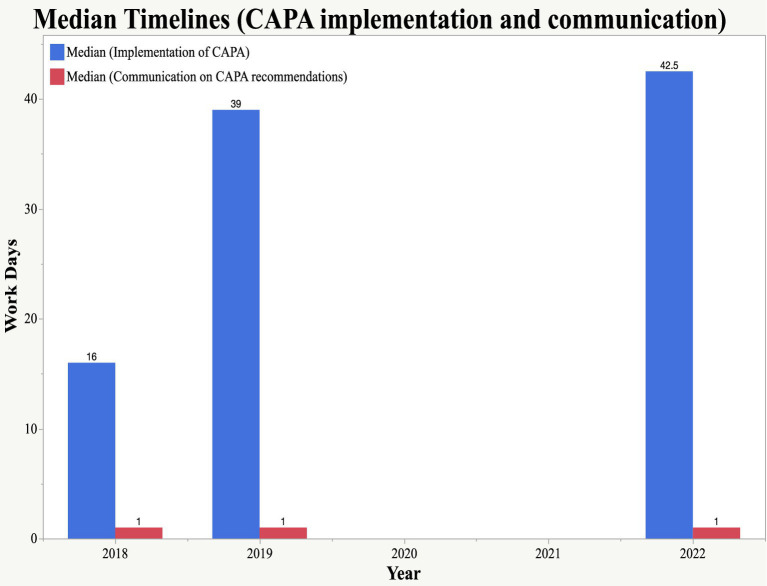
Median timelines for CAPA implementation and communication on outcomes of CAPA review.

Figure 7 summarizes the findings of the timelines for the implementation of CAPA by the applicant and communication by the NDA to the applicant on the outcomes of the CAPA review. Based on these findings, both applicants implemented the CAPA within 90 workdays. Median timelines of 16 days (2018), 39 days (2019), and 42 days (2022) were observed. Communication to applicants by the NDA concerning the outcomes of the CAPA review was done within a timeframe of 5 days. Communication by the NDA was done on time as per the findings, which indicated a median time of 1 day throughout the years.

### 3.6 Relevance of the data to the current regulatory ecosystem

While the analysis in the manuscript covers data up to 2022, the findings remain highly relevant in 2025 for several reasons:

1. Timelines as a Benchmark: The inspection timelines analyzed provide a valuable benchmark for assessing progress and identifying persistent bottlenecks in the joint EAC GMP inspection process. These benchmarks are essential for evaluating improvements or regressions in regulatory efficiency over time.
2. Capacity Building Trajectory: The trends observed up to 2022 offer insights into the trajectory of capacity building among less-resourced National Regulatory Authorities (NRAs). Many of the systemic challenges and capacity gaps identified remain pertinent, and the recommendations proposed continue to inform ongoing efforts to strengthen regulatory systems.
3. Policy Continuity and Implementation: Regulatory reforms and capacity-building initiatives often extend over several years. The data and analysis from 2022 provide a foundation for understanding the impact of policies implemented since then and for guiding future interventions.
4. Limited Availability of More Recent Data: As of now, comprehensive and validated data beyond 2022 on joint EAC GMP inspections is limited. Therefore, the 2022 dataset remains the most robust and complete source for drawing meaningful conclusions.

## 4 Discussion

Optimizing EAC joint regulatory activities and continuously building the capacity of less-resourced NRAs is key to ensuring the full potential of harmonization and regulatory system strengthening in Africa (23). The assessment of timelines for EAC joint GMP inspections is important for evaluating the value addition of the procedure to access quality medicines, cost reduction, and duplication of efforts in the region (18, 19, 24). Additionally, the findings from this study will inform areas of regulatory system strengthening and capacity building at the regional and continental levels. Capacity building at the continental level can be through technical committees of the AMA, specifically GMP technical committee (GMP-TC), regulatory capacity building technical committee (RCD-TC), and information management system technical committee (IMS-TC).

The timeline for screening the application and master file was 5 days. The findings indicated that a timeline of 7 days was recorded in 2020, whereas the lowest median time of 2 days was recorded in 2017. The COVID-19 pandemic, which impacted all sectors, including the EAC joint GMP inspections, contributed to a long median time. Joint inspections were suspended due to travel restrictions and limited operations by pharmaceutical industries and regulatory agencies. To maintain an efficient and functioning regulatory environment, agile and flexible approaches such as remote inspections have been introduced in some countries (25). Alternative inspections were used in the hybrid model, which included a remote/virtual desktop review of information and inspection, and *in situ* inspections under controlled conditions (4). However, owing to limited infrastructure and technology, the EAC region could not implement remote or hybrid inspections.

The median time for scheduling joint EAC GMP inspections was longer than 14 days throughout the study period: 57 days (2018), 24 days (2019), 43 days (2020) and 28 days (2022). Tentative scheduling is performed by the NDA; nonetheless, consensus must be reached at the regional level to avoid conflicts with the calendars of individual participating NRAs. At the regional level, either a virtual or face-to-face expert working group for GMP is convened by the EAC Secretariat to review the tentative schedule and plan for joint GMP inspections and ensure that consensus is reached by inspectors of each EAC NRAs before joint physical inspection or desk assessment of documentation is conducted. The meeting also provided the opportunity to map common applications for GMP inspection in each EAC NRA. Subsequently, the identified common sites pending inspection are included in the plan for joint inspections. Based on these findings, there is a need for the region to establish quarterly scheduling and planning meetings to ensure timely mapping of common applications and build a consensus on the schedule of joint inspections based on the applications received by the NDA. To make the process more transparent, scheduling dates can be planned on a yearly basis and communicated to all the EAC NRAs to avoid conflicts with other regional or national activities.

Planning for joint GMP inspection was consistent with the set timelines of 14 days, except in 2017, when the median timelines of 40 days were observed. The main reason for this is that the region was in the initial stage of piloting the EAC-MRH program, which included the domestication of a harmonized regulatory framework for GMP. Improvements were observed in the median timelines for the planning phase of joint inspections, which were reduced to 20 days in 2018, 18 days in 2019, 28 days in 2020, and 5 days in 2022. This has been attributed to the timely coordination, planning, and convening of GMP expert working group meetings by the EAC Secretariat.

Communication with applicants on schedules and plans for joint inspections and, in most instances, seeking consent from applicants to conduct regional joint inspections instead of individual NRAs inspections have been maintained within the timeframe. The median time for communication with applicants was maintained consistently for 1 day in 2016, 2017, 2018, 2019, and 2022. In 2020, the median time of communication by the NDA to applicants was 2 days, although joint inspections were not conducted. Similarly, communication by the NDA to EAC NRA’s was recorded to have a median timeline of 1 day throughout the years, as indicated in Figure 5b NDA has ensured consistency in communication with both applicants and EAC NRAs, which is key for smooth execution of joint inspections.

Joint physical inspections have been conducted within the time frame of 5 days. The maximum median time was 3 days, which was recorded in 2016, 2018, and 2022, whereas the lowest median time was 1 day, which was observed in 2017. For 2018, two desk reviews of GMP documentation were conducted; hence, the median timelines recorded in 2018 were factored into the desk assessment of GMP documentation. However, the metric tool did not have data for the eight desk assessments conducted in 2022 for applications that met that criterion. The completeness of the metric tool and capture of the timelines for physical inspections and desk assessment will inform the participating NRAs and the EAC Secretariat of areas that need improvement in systems or approaches, policies, guidelines, or capacity building of inspectors.

Furthermore, joint physical inspections, which are normally conducted by three inspectors, have been used for the hands-on training of less-resourced NRAs. Two inspectors are drawn from mature NRAs, while one inspector is drawn from a less-resourced NRA. In addition, technical teams from the EAC Secretariat were involved in physical inspections. Fewer-resourced NRAs have continued to benefit from joint inspections and assessments of medicinal product dossiers. In addition to hands-on experience through joint physical inspections, basic and advanced GMP training for experts in EAC NRAs was conducted by the EAC Secretariat in collaboration with the World Health Organization (WHO) (14). The GMP training modules included theoretical and practical sessions. It has been almost a decade since the commencement of the joint physical inspections and capacity-building programs within the framework of the EAC-MRH program, and there is a need to evaluate the impact of physical joint inspections as a tool/model to build the knowledge, skills, and competency of inspectors from less-resourced NRAs. Similarly, the evaluation of the EAC joint assessment procedure as a capacity-building model for less-resourced NRAs should be explored.

Median timelines for report writing following physical inspection have been maintained within the time frame of 14 days, except in 2022, where the median time was 16 days. The median timelines for peer review of the report by all EAC inspectors were 16, 33, and 15 days in 2018, 2019, and 2022, respectively. The metric tool does not contain data from 2017. The main reasons for long median timelines for peer review include delays in writing report by inspectors, review process takes time due to contributions/inputs made by all EAC inspectors on improvements to be made to the report (which add more time for peer review), non-availability of inspectors to present the report during virtual peer review meetings and time to address addional inputs before reaching consensus on the final report. The EAC has developed a harmonized inspection report template; nevertheless, delays in writing may be attributed to competency in preparing inspection reports. Uche et al. retrospectively reviewed the competency of inspectors in writing GMP inspection reports in West Africa. A review of 25 GMP reports revealed that 4% of the reports qualified as excellent report,8% as good reports, and 44 reports as requiring improvement or unacceptable reports (26).

The research findings indicated timely communication to applicants on the outcomes of peer review meetings, while long median timelines for the submission of CAPA by applicants were 135 days and 125 for the year 2018 and 2019, respectively. A decrease in timelines for the submission of CAPA by an applicant was observed in 2022, with a median timeline of 49 days. A review of CAPA by EAC inspectors must be performed within 14 days. Based on the findings, this milestone was achieved within the timelines of 13 days, 3 days, and 1 day in 2018, 2019, and 2022, respectively. The results indicate the need for continuous capacity building for pharmaceutical manufacturers to ensure compliance with GMP.

The analysis further revealed that the timeframe for the implementation of CAPA by the applicants was within 90 days in 2018, 2019, and 2022. Facilities that were jointly inspected in 2017 were found to be compliant with the EAC GMP standards (they were not required to submit CAPA) and were issued GMP certificates, which were valid for 3 years. Two facilities inspected in the year 2016, they have not yet submitted CAPA until to date. During COVID-19 pandemic in 2020 and 2021, facilities which were due for routine physical inspections and renewal validity of their GMP certificate, were given an extension of 1 year since physical inspections could not be conducted.

The findings of this study have shown that the EAC region has continued to embrace integration, specifically in the strengthening, harmonization, convergence, and reliance of the medical product regulatory system, which has built trust and confidence among inspectors. Additionally, joint EAC GMP inspection continues to be a tool/model for capacity building of less-resourced NRAs. Twinning between EAC NRAs has provided an opportunity for less resourced NRAs to learn and acquire practical skills from advanced EAC NRAs in areas of inspection covering the 5Ps—Products, People, Processes, Procedures, and Premises—for API and sterile and non-sterile manufacturing facilities. Building the competency and skills of staff in regulatory agencies is a prerequisite for enforcing regulations and ensuring that medicinal products meet safety, efficacy, and quality standards. It also builds the competency of regulators in educating communities and contributes to advancements in research, innovation, and development.

Studies have indicated skill gaps in regulatory sciences that require a comprehensive strategy for sustainable training and professional development on the African continent (27, 28). The development of new innovative biotechnological products, advancement in medical devices, and information technology sectors need to match skilled human resources in line with the WHO competency framework for regulators (29). The mapping of regulatory science training programs offered by different higher learning institutions located within and outside the continent to provide capacity building to both regulators and the pharmaceutical industry is crucial for addressing skills and competency gaps in Africa (30–33).

Furthermore, the EAC joint GMP inspection model should be adopted by the Secretariat of the African Medicines Agency (AMA) through the continental technical committees for GMP and capacity building to ensure the contribution of all NRAs in safeguarding the public health of the people of the continent. To ensure that safety, efficacy, and quality medicines, vaccines, and health technologies are accessible to those in need, NRAs require human resources with the right skills, knowledge, and competency to implement all eight regulatory functions, as outlined in the WHO Global Benchmarking Tool (34). Additionally, the EAC joint regulatory inspection process should be optimized and gaps identified should be addressed, including incompleteness of the data in the metric tool, delays in the submission of CAPA by applicants, and minor delays in report writing and peer review of the inspection report.

More studies are required to quantify the impact of EAC joint GMP inspections and joint assessment of medicinal product dossiers as a capacity-building tool/model for less-resourced NRAs. Research studies can also examine strategies implemented by less-resourced NRAs to ensure that the skills, knowledge, and competencies gained are transferred and retained within their institutions.

As the world continues to advance into new innovative medical products, vaccines, and technologies, including the use of artificial intelligence (AI), AMA will need to identify, support, and collaborate with high-learning institutions (within and outside the continent) to ensure the continuous provision of capacity building for pharmaceutical personnel, strengthening research, and domestic pharmaceutical production.

### 4.1 Recommendations for strengthening GMP assessment and regulatory collaboration

- Update the metric tool to include data from desk assessments of GMP documentation and records conducted during previous regulatory activities, ensuring historial data is captured for trend analysis and process improvement.
- Establish and monitor milestones for each step of joint physical inspections and desk assessments of GMP documentation. Implement timely monitoring of each milestone and record timelines using a clock-stop system to reduce delays and improve access to quality-assured medical products
- Strengthen regional capacity to implement alternative inspection methods, such as remote regulatory assessment (RRA) to ensure continuity of GMP compliance evaluations during public health emergencies or other disruptions.
- Leverage advancements in information technology to develop or enhance metric tools that promote consistency, completeness, and accuracy in data entry and reporting across regulatory agencies.
- Enhance stakeholder feedback mechanisms to minimize delays in the submission of corrective and preventive actions (CAPA) from facilities, thereby shortening timelines for regulatory inspections and decision-making.
- Support pharmaceutical manufacturers in building and maintaining GMP compliance capacity to address CAPA efficiently and effectively, contributing to improved product quality and regulatory outcomes
- Promote knowledge exchange and capacity building through joint regulatory activities, such as product assessments and inspections, to strengthen the practical skills and competencies of staff in less-resourced National Regulatory Authorities (NRAs).

## 5 Conclusion

This is the second study to analyze the timelines of the EAC-MRH program. The first study focused on the joint assessment procedure of medicinal product dossiers, whereas this study analyzed the timelines for the EAC joint inspection of pharmaceutical manufacturing facilities to verify compliance with current GMP (cGMP). The findings indicated adherence to set timelines for screening applications and master files, conducting joint physical and desk assessments of GMP records,communication between NDA and applicants and NRAs, and review of CAPA. However, the median timelines for scheduling joint inspections were observed to be more than 14 days throughout the years (2016–2022), and a regional consensus on the dates for NRAs to conduct joint inspections needs to be reached and confirmed by all EAC NRAs to avoid conflict with national activities. The study further revealed that the median timelines for report writing following physical inspection were maintained within a timeframe of 14 days, except in 2022, where the median time was 16 days. The median timeline for peer review of the report by all EAC inspectors was more than 14 days, and the main reasons were delays in writing reports and/or the incorporation of regional inputs into the draft report. The metric tool had missing data on peer review milestones in 2017. Long median timelines were also observed for the submission of CAPA by an applicant between 2018 and 2019, which was more than the agreed timeline of 90 days, and during the year 2022, the timelines were reduced to 49 days. Strengthening the capacity of pharmaceutical manufacturers to comply with GMP standards will reduce the issuance of CAPA by regulators, and subsequently reduce the timelines for joint inspection procedures. Identified gaps in the metric tool such incompleteness of data and missing data for a procedure of desk assessment of GMP records needs to be addressed to ensure future analysis of timelines reflect physical and abridged EAC joint GMP inspections.

### 5.1 Limitations

The metric tool does not have data for the eight (8) desk assessment of the GMP records conducted in 2022. Eight participating NRAs—ABREMA, DFCA, NDA, PPB, Rwanda FDA, TMDA, and ZFDA—were involved in EAC joint regulatory inspections. The NRAs of the DRC and the Republic of Somalia were not considered because the two countries joined the East African Community in July 2022 and March 2024, respectively.

## Data Availability

The original contributions presented in the study are included in the article/supplementary material, further inquiries can be directed to the corresponding author.
